# The role of primary surgical treatment in young patients with squamous cell carcinoma of the larynx: a 20-year review of 34 cases

**DOI:** 10.1186/s12957-015-0699-y

**Published:** 2015-09-24

**Authors:** Junxi Wang, Xingguo Zhao, Xinliang Pan, Limin Zhao, Jianming Zhou, Min Ji

**Affiliations:** Department of Otolaryngology-Head and Neck Surgery, Affiliated Hospital of Weifang Medical College, 2428 Yunhe Road, Weifang, 261031 Shandong People’s Republic China; Department of Otolaryngology-Head and Neck Surgery, Qilu Hospital, Shandong University, Jinan, 107#, Wenhua Xi Road, Jinan, Shandong 250012 People’s Republic of China

**Keywords:** Young patients, Larynx, Squamous cell carcinomas, Survival, Laryngectomy, Prognosis

## Abstract

**Background:**

The aim of this study was to investigate the clinical patterns in young Chinese patients (less than 40 years old) with laryngeal squamous cell cancer (LSCC) and the outcome of primary open surgery.

**Methods:**

Thirty-four young patients, with histologically confirmed LSCC between 1985 and 2005 at Qilu Hospital and Affiliated Hospital of Weifang Medical College, who underwent primary open surgery were retrospectively evaluated according to the clinical patterns in comparison with 374 non-young patients (older than 40 years). The Kaplan-Meier method was used to calculate the survival rate. The relevance of smoking, tumor location, tumor-node-metastasis (TNM) staging, lymph node involvement, tumor size, and histological differentiation to overall survival was tested by multivariate analysis.

**Results:**

There was a significantly higher rate of smoking (*p* = 0.020) in the non-young patients compared to the young patients, but no significant difference was observed in alcohol consumption, tumor location, tumor size, TNM staging, lymph node metastasis, histological grade, and 5-year overall survival.

One-year survival rates were 100 %, 3-year survival rates were 79.41 %, and 5-year survival rates were 67.65 %. In the multivariate analysis, lymph node involvement (*p* = 0.006), tumor stage (*p* = 0.022), and tumor size (*p* = 0.004) proved to be significant predictors of overall survival.

**Conclusions:**

The incidence of smoking was significantly higher in non-young patients compared to young patients. Primary surgery with or without radiotherapy may provide a value treatment option for young LSCC. Nodal status, tumor stage, and tumor size were the primary determinants of overall survival in multivariate analysis. These data may provide useful information for counseling and treatment planning.

## Background

Laryngeal carcinoma, which originates from a subsite of the upper respiratory tract, was diagnosed in 20,875 men and women in China in 2011 [1], and laryngeal squamous cell carcinoma (LSCC) is by far the most frequent histology. As this type of cancer impairs the functions of breathing, swallowing, and speech, treatment decisions for this disease are dependent in large part on assessment of disease extent and performance status with the aim of high-survival rates and excellent organ function preservation. Great developments in management of laryngeal carcinoma have been seen over the past century. Surgery, radiotherapy, and chemotherapy alone or in combination are the main treatments for this disease.

The standard of management for laryngeal carcinoma still remains controversial. It is hard to justify, from the literature, which choice of therapy is the best. Partial laryngectomy, laser resection, or radiation therapy alone is utilized for early laryngeal tumors, while subtotal laryngectomy or total laryngectomy followed by radiotherapy is adopted for advanced laryngeal malignancies [2]. However, the Department of Veterans Affairs Laryngeal Cancer Study Group offered the potential value of induction chemotherapy plus radiation in achieving laryngeal preservation with survival rates comparable to surgery as the primary treatment for advanced laryngeal cancer [3]. Of note, a recent study indicated that laryngeal carcinoma survival has decreased among patients with laryngeal cancer during the past two decades in the USA [4]. More recently, several findings involving the therapeutic use of surgery showed that this treatment might be a better therapy for better survival outcomes than nonsurgical therapy for patients with advanced laryngeal cancer [5, 6]. Although there is no difference in choice of treatment between the young and non-young patients with LSCC, the experience of managements of these young patients with LSCC is so limited in that young patients less than 40 years old are relatively rare [7, 8].

To date, few reports have been available focusing on clinical characteristics of young LSCC and the efficacy of open surgery as the initial treatment for them. With these points in mind, our aim was to investigate the clinical characteristics of young patients with LSCC compared with older counterparts and to evaluate the value of open surgery. Moreover, we further explored the prognostic factors that had a significant impact on 5-year overall survival rates.

## Methods

A total of 34 young Chinese patients with LSCC (less than 40 years) who underwent partial, subtotal, or total laryngectomy as the initial treatment in Qilu Hospital and the Affiliated Hospital of Weifang Medical College between the years 1985 and 2005 were enrolled in our study. Another 374 LSCC patients aged over 40 years were randomly selected as a comparison cohort. The patterns of clinicopathological and 5-year survival rates after treatments were compared between the two groups.

Those patients with distant metastases or with histological findings other than squamous cell carcinoma at the time of diagnosis were excluded from the study. None of these patients had received preoperative radiotherapy or adjuvant therapy, and none were treated by transoral laser surgery. High-resolution computed tomographic (CT) scanning of the primary tumor was performed to ascertain extent of the tumors.

The diagnoses were confirmed by a combination of laryngoscopic examination and preoperative pathological examination. The histological specimens in each case were examined by two competent pathologists without knowledge of the patients’ clinicopathological characteristics and clinical outcome. Discrepancies were resolved by two advanced pathologists who reviewed the immunostained slides together under a multi-head microscope until a consensus was reached. Furthermore, the histological subtypes were determined by analyzing the specimen slides. Clinical stages were determined according to the tumor-node-metastasis (TNM) classification system of the International Union [9].

After successful resection of the tumor with an electric knife, the specimens were sent for final histopathological evaluation. Moreover, the surgical margin specimens are rapidly submitted to a pathologist for careful frozen histopathological section examination. If positive, additional tissue was removed until the margins were clear in order to reduce postoperative recurrence of the carcinoma.

Selective neck dissection was performed for N0 neck with supraglottic cancers. Bilateral modified neck dissection is commonly performed for cases with clinically evident neck nodal metastases.

Postoperative anti-inflammatory drugs and dressing changes each day were utilized for about 8 days. Nutritional support was applied due to abrosia, and the average period of time before the nasogastric tube was removed was 10.33 days. Postoperative radiotherapy was carried out 5 weeks after the operation when necessary. The mean time of hospital stay was 15.36 days.

The patients were followed up every 3 months in the first year after operation, every 6 months in the second year, and every 2 years after the operation. The follow-up ended in April 2013. The overall survival was defined as the internal between the date of surgery and the date of death. The median follow-up period after surgery was 62.52 months (range 13–120 months).

### Statistic analysis

The chi-square test was used to analyze the correlation between clinicopathological characteristics of the two groups. Kaplan-Meier survival curves were employed to evaluate the 5-year overall survival. The log-rank test was used to draw survival curves. A multivariate Cox regression model was adopted to evaluate the independent effect of clinicopathological features diagnosis on survival from the younger patients with LSCC. All statistical tests were two-sided, and *p* values less than 0.05 were considered to be statistically significant. All analyses were conducted using SPSS version 16.0 statistical software

## Results

**Fig. 1:**
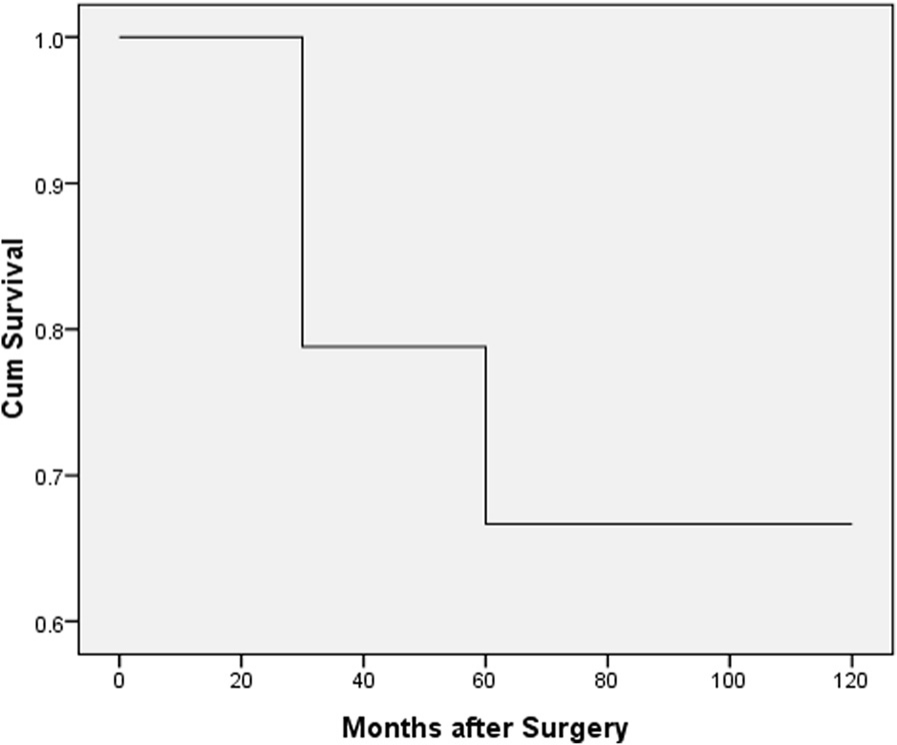
A Kaplan-Meier analysis of the overall survival of young patients with LSSC

The total of 34 patients that satisfied the inclusion criteria were analyzed as the younger age group. Among these, the mean age was 34.6 2 years and the range was from 24 to 39 years. A detailed description of age is presented in Table 1. All 34 patients were male. When classified according to anatomic location, 11 cases (32.35 %) had supraglottic carcinoma, 22 cases (64.71 %) had glottic carcinomas, and one case (2.94 %) had infraglottic carcinoma. According to pathology, 24 cases (70.59 %) were classified as well and moderate differentiated and ten cases (29.41 %) as poorly differentiated. For younger patients, the most common operation was vertical partial laryngectomy with 21 surgeries (61.76 %), followed by eight horizontal glottis surgeries (23.53 %), four (11.76 %) total laryngectomy, and one (2.94 %) subtotal laryngectomy. Two patients, one with positive and one with close surgical margins underwent surgical re-excision. Fourteen patients (41.18 %) also had a nodal operation. Postoperative radiotherapy was recommended to optimize regional control for 15 (44.12 %) patients. The functional larynx was successfully preserved in 29 (85.29 %) of the younger patients. Decannulation was performed in 28 (82.35 %) younger patients. As for postoperative complication, one patient experienced infection and no patients died. For the younger population, 1-year survival was 100 % in this series, 3-year survival was 79.41 %, and 5-year survival was 67.65 %, as shown in Fig. 1.

**Table 1:** Presentation of age in cases of young patients

Characteristic	Number of patients (%)
Age (years)	
0–20	0
21–25	1 (2.94)
26–30	2 (5.88)
31–35	8 (23.53)
36–39	23 (67.65)

With regard to the 374 patients diagnosed with LSCC (older than 40 years), their mean age was 58.61 years ranging from 40 to 85 years. Of these patients, 329 (87.97 %) were male and 45 (12.03 %) were female. When classified according to anatomic location, 82 cases (21.93 %) had supraglottic carcinomas, 278 cases (74.33 %) had glottic carcinomas, and 14 cases (3.74 %) had subglottic carcinomas. According to pathology, 301 cases (64.97 %) were classified as well differentiated and moderate, and 73 cases (33.96 %) as poorly differentiated. The clinical stage distribution according to age group was as follows: stage I and stage II, 21 (61.76 %) patients in the younger age group and 247 (66.04 %) in the older age group, while 13 (38.24 %) cases were found in stage III and stage IV in the younger age group and 127 (33.96 %) cases in the older age group. For the older LSCC patients, 1-year survival, 3-year survival, 5-year survival were 100, 75.68, and 66.31 %, respectively. A detailed description of sex, tumor localization, N status, histological differentiation, and other demographics is presented in Table 2. There was a significantly higher rate of smoking (*p* = 0.020) in the non-young patients compared to the young ones, but no significant difference was observed in alcohol consumption, tumor location, tumor size, TNM staging, lymph node metastasis, histological grade, and 5-year overall survival.

**Table 2:** Clinicopathological variables between the less than 40 years group and the more than 40 years group

Variables	No. of patients		*p*
Age (years)	<40	≥40	
Alcohol consumption			
Current or former	20 (58.82 %)	260 (69.52 %)	0.198
Never	14 (41.18 %)	114 (30.48 %)	
Smoking			
Current or former	20 (58.82 %)	287 (76.74 %)	0.020
Never	14 (41.18 %)	87 (23.26 %)	
Tumor location			
Larynx glottic	22 (64.71 %)	278 (74.33 %)	0.223
Larynx non-glottic	12 (35.29 %)	96 (25.67 %)	
Histological differentiation			
Well and moderate	24 (70.59 %)	301 (80.48 %)	0.170
Poor	10 (29.41 %)	73 (19.52 %)	
Tumor size			
Less than 3 cm	25 (73.53 %)	276 (73.80 %)	0.973
More than 3 cm	9 (26.47 %)	98 (26.20 %)	
Node involvement			
Yes	6 (17.65 %)	76 (20.32 %)	0.710
No	28 (82.35 %)	298 (79.68 %)	
Stage			
I + II	21 (61.76 %)	247 (66.04 %)	0.615
III + V	13 (38.24 %)	127 (33.96 %)	
5-year survival			
Alive	23 (67.65 %)	248 (66.31 %)	0.874
Dead	11 (32.35 %)	126 (33.69 %)	

*p* value is the result of chi-square test in the same factor, and *p* < 0.05 is considered statistically significant

Multivariate analysis was used to analyze prognostic factors of 5-year overall survival (OS) for patients. As depicted in Table 3, some variables were stratified to assess the different effects on OS. In addition to lymph node status (*p* = 0.006), TNM stage (*p* = 0.022) and tumor size (*p* = 0.004) were independent prognostic factors for poorer OS in multivariate analysis. However, no statistically significant correlation between smoking (*p* = 0.602), differentiation status (*p* = 0.154), tumor location (*p* = 0.324), and overall survival was found among the young patients with LSCC.

**Table 3:** Multivariate analysis of 5-year survival in young Chinese LSCC patients after surgery

Factors	B	SE	Wald	Sig	Exp (B) (95 % CI)
Smoking (current or former/never)	1.788	1.171	2.331	0.127	5.976 (0.602–59.298)
Histologic grade (non-poor/poor)	−0.390	0.755	0.266	0.606	0.677 (0.154–2.976)
Tumor location (larynx, glottic/larynx, nonglottic)	0.528	0.844	0.390	0.532	1.695 (0.324–8.868)
The largest size (≤3 cm/>3 cm)	−3.350	1.152	8.460	0.004	0.035 (0.004–0.335)
TNM stage (I + II/III + IV)	−2.442	1.066	5.245	0.022	0.087 (0.011–0.703)
Lymph node metastasis (absent/present)	−3.148	1.150	7.492	0.006	0.043 (0.005–0.409)

## Discussion

The study data consisted of cancer patients over a long term from 1985 to 2005 and in a wide area including large rural areas and urban areas. As such, this report represents a view of the successful management of younger patients with laryngeal SCC.

The incidence of head and neck cancer differed in various regions. A previous report showed that the incidence of head and neck cancer was increasing in women [10]. Also, the incidence of women appeared to increase in The Netherlands while the male to female ratio of this disease has declined [11]. For the total of Chinese patients, the male to female ratio of laryngeal carcinoma is commonly about 6–7:1, but it is about 1.97:1 in the northeast of China, maybe partly due to more female smokers in this region [2]. In the present study, there were no female laryngeal cancer patients in the younger cohort.

With regard to the development of laryngeal carcinoma, it is a complex process that involves multiple steps. Of the factors affecting the progress of laryngeal cancer, smoking may play a significant role in the progress. In this present investigation, 58.82 % of young patients had a history of heavy smoking. The rate of smoking was significantly higher in non-young patients compared to young patients, which is in agreement with a previous study [12].

Cancer of the larynx is divided anatomically into glottis carcinoma, supraglottic carcinoma, and subglottic carcinoma. In the present study, over one third of the younger patients had primary supraglottic squamous cell carcinoma, and nearly two thirds of the younger patients had primary glottic squamous cell carcinoma. Patients with glottic squamous cell carcinoma are usually diagnosed in an early stage and have an excellent prognosis. Optimal treatment for LSCC has not been established, and treatment options have been dependent on the institution’s policy. Our preference has been to use open partial laryngectomy for the majority of the patients. The surgeon should consider the patient’s prognosis with the preservation of postoperative function. Also, the surgery of laryngeal cancer should be individualized according to the size and extent of the tumor, the age, and physical condition of the patient, and the skill and experience of the surgeon with various treatment modalities and surgical procedures [13].

Open partial laryngectomy which is important in the management of patients with early LSCC has already been characterized. First, it is believed that open partial laryngectomy preserves the organ function to increase the quality of life. Second, in contrast to radiotherapy, open surgery can be repeated for curative purposes, whereas postoperative radiation therapy in patients with positive margins did not improve survival [14].

Third, patients with T2 tumors may have a better local control rate after open partial laryngectomy compared to laser resection and radiotherapy [15].

In the young cohort, we had a high number of open partial laryngectomy (29 patients or 85.29 %), with good functional larynx preservation (30 patients or 88.24 %) and high decannulation which improved the quality of life. The 5-year survival rate was 67.65 %, which is similar to a previous study [4].

The major prognostic factors for head and neck cancer are the presence of lymphatic involvement, positive surgical margins, locoregional metastasis, T stage, and extracapsular spread of tumor cells from involved nodes into the soft tissue of the neck [16, 17]. Another study showed that young age may confer a worse prognosis in patients with squamous cell carcinoma of larynx [5]. In the present study, our analysis identified that nodal status, tumor stage, and tumor size adversely impacted 5-year overall survival.

The strengths of the study were that it featured adequate follow-up, a comparison with a large cohort, and face-to-face conversation with patients or their relatives to obtain detailed information. However, future surveys with larger study cohorts are warranted to further verify our results.

## Conclusions

To conclude, the findings presented here are the first to investigate the clinical patterns in young Chinese patients with LSCC and their outcome of open surgery. This study has shown that the incidence of smoking was significantly higher in non-young patients compared to young patients and that open surgical treatment as the initial treatment was effective for the young cohorts. In addition, nodal status, tumor stage, and tumor size were the primary determinants of 5-year overall survival. These data may provide useful information for counseling and treatment planning for anti-laryngeal cancer therapy.

### Ethical approval

This study has been approved by the Ethics Committee of the Qilu Hospital of Shandong University Medical College and Affiliated Hospital of Weifang Medical College and was performed in accordance with the ethical standards laid down in the 1964 declaration of Helsinki and all subsequent revisions. All persons mentioned in the paper gave their informed consent prior to their inclusion in the study.

## Abbreviations

LSCC: laryngeal squamous cell cancer
OS: overall survival
